# Investigation of Antimicrobial and Anti-Inflammatory Efficacy of Newly Synthesized Pyrogallol-Coumarin Hybrids: *In Vitro* and *In Silico* Studies

**DOI:** 10.3390/pharmaceutics16111472

**Published:** 2024-11-18

**Authors:** Dušica Simijonović, Edina Avdović, Sandra Jovičić Milić, Marko Antonijević, Dejan Milenković, Katarina Marković, Mirjana Grujović, Danijela Lj. Stojković, Milan Dekić, Zoran Marković

**Affiliations:** 1 Department of Science, Institute for Information Technologies, University of Kragujevac, Jovana Cvijića bb, 34000 Kragujevac, Serbia; dusicachem@kg.ac.rs (D.S.); sandra.jovicic@pmf.kg.ac.rs (S.J.M.); mantonijevic@uni.kg.ac.rs (M.A.); dejanm@uni.kg.ac.rs (D.M.); katarina.mladenovic@pmf.kg.ac.rs (K.M.); mirjana.grujovic@pmf.kg.ac.rs (M.G.); danijela.stojkovic@kg.ac.rs (D.L.S.); zmarkovic@np.ac.rs (Z.M.); 2 Department of Natural Science and Mathematics, State University of Novi Pazar, Vuka Karadžića 9, 36300 Novi Pazar, Serbia; mdekic@np.ac.rs; 3 Department of Engineering and Natural Sciences, University of Applied Sciences Merseburg, Eberhard Leibnitz-Str. 2, 06217 Merseburg, Germany

**Keywords:** pyrogallol-coumarin hybrids, anti-inflammatory effect, antimicrobial activity, molecular docking, molecular dynamics simulations

## Abstract

**Background:** The aim of this study is to present the synthesis of two new compounds with promising antimicrobial and anti-inflammatory properties using precursors that contain pyrogallol and coumarin units. **Methods:** The characterization of the obtained compounds (**PCHs**) (*E*)-*N*′-(1-(2,4-dioxochroman-3-ylidene)ethyl)-2,3,4-trihydroxybenzohydrazide (**PCH-1**) and (*E*)-*N*′-(1-(2,4-dioxochroman-3-ylidene)ethyl)-3,4,5-trihydroxybenzohydrazide (**PCH-2**) was performed using various spectroscopic methods in combination with the DFT methods. To evaluate antimicrobial and anti-inflammatory activities, **PCHs** were tested against 13 different types of microorganisms and soybean lipoxygenase. To determine the specific mechanisms of anti-LOX activity, molecular docking and molecular dynamics studies were performed. **Results:** These compounds had the most potent antibacterial activity against the bacterium *Proteus mirabilis* ATCC 12453, with a MIC value of 31.125 µg/mL. In addition, three standard bacterial species were chosen to evaluate the antibiofilm activity of tested substances. The results showed that the strongest effect of **PCH-2** was noticed on the biofilm formation of *Staphylococcus aureus* ATCC 25923 (BIC_50_ at 378 µg/mL). The anti-LOX results indicate that **PCHs** have excellent activity with the IC_50_ value for **PCH-1** = 38.12 μM and **PCH-2** = 34.12 μM. **Conclusions:** The obtained in vitro and in silico results confirm the strong inhibitory potential of the investigated compounds.

## 1. Introduction

Benzene-1,2,3-triol, also known as pyrogallol, is a highly potent reducing agent that is found abundantly in nature [1]. This compound is present in a variety of fruits, vegetables, tea leaves, and nuts. It is renowned for its antioxidant capabilities and has been extensively researched for its possible health advantages. Thanks to these properties, it is used as a chemical precursor in the synthesis of pharmacologically important compounds. Additionally, pyrogallol has been employed in the formulation of hair dyes, as well as in the manufacturing processes of ink and rubber. Due to its wide range of applications, pyrogallol is very adaptable and has great importance both in the pharmaceutical and in numerous other industries [1].

The coumarin nucleus is a bicyclic heterocycle consisting of fused γ-pyrone and benzene rings, which have a carbonyl group in position 2 of the pyrone ring. They form a class of natural compounds that are found in some essential oils (lavender oil and cassia leaf oil), as well as certain types of cinnamon [2]. In addition to being found in all parts of the plants (root, stem, and leaf) [3,4,5,6], they also have been found in some microorganisms, such as aflatoxins found in the *Aspergillus* species, as well as antibiotic novobiocin, isolated from *Streptomyces *sp. The presence of coumarins in both natural sources and microorganisms highlights their diverse roles in different biological systems. These compounds have also shown potential in the treatment of various diseases, such as cancer and cardiovascular diseases [3,4,7,8,9,10,11]. Apart from naturally occurring variations, there currently exists a great array of coumarin molecules that are synthesized in laboratories. Coumarin derivatives, whether natural or synthetic, have demonstrated a broad spectrum of biological activity, including anticoagulant, anti-inflammatory, and antibacterial characteristics [12,13,14,15]. Overall, the diverse range of biological activities exhibited by coumarins makes them a valuable area of study for potential therapeutic interventions [16,17,18].

A class of iron-containing enzymes known as lipoxygenases (LOXs) catalyzes the deoxygenation of polyunsaturated fatty acids in lipids with a *cis*,*cis*-1,4-pentadiene structure. They transform arachidonic acid, a component of membrane phospholipids, into pro-inflammatory mediators known as leukotrienes [19,20]. These molecules are a class of extremely potent compounds with a wide range of biological actions, so the development of LOX inhibitors for medicinal reasons has attracted a lot of attention lately [21]. Several naturally occurring coumarins, such as esculetin, fraxetin, and daphnetin, are known as inhibitors of the pro-inflammatory process, i.e., lipogenesis [22]. Studies have shown promising results in reducing inflammation and pain, indicating that coumarin derivatives may be a valuable option for managing inflammatory diseases in the future [11,23]. Research on the anti-inflammatory activity of coumarin derivatives continues in order to reveal their full potential in the treatment of inflammatory conditions. In addition, another huge health problem in the world is infectious diseases. Some coumarins, such as novobiocin, umbelliferone, and scopoletin, have been shown to exhibit excellent antibacterial and antifungal activity [24,25]. Coumarin derivatives with excellent antimicrobial properties have the potential to be used in the development of new antibiotics and antifungal agents due to their natural origins and effectiveness against various pathogens.

Considering that some synthetic and natural derivatives of coumarin have found themselves in clinical use, these compounds become a subject of research by many scientists [3,4,7,8,9,10,11]. Our research group has been studying the biological properties of many coumarin derivatives for an extended period of time, including their antioxidant, anticancer, antibacterial, and antifungal activity [12,16,17,18]. We have found that certain coumarin derivatives exhibit promising potential in inhibiting the growth of cancer cells in vitro [16].Furthermore, our research has revealed that some of these compounds have substantial antioxidant and antibacterial activities, making them promising candidates for pharmaceutical development [12,16,17,18]. The purpose of this study is to evaluate the anti-inflammatory, antibacterial, and antifungal activity of newly synthesized pyrogallol-coumarin hybrids (**PCH**s). Information on anti-inflammatory and antimicrobial properties could provide significant insight into their potential pharmaceutical applications. The precise mechanism by which these compounds exert their inhibitory effects, particularly through interactions within the LOX active site, remains to be elucidated. This study utilizes both experimental and computational approaches, including molecular docking and molecular dynamics (MDs) simulations, to explore the LOX inhibitory potential of these hybrids and investigate their interactions at the molecular level. Understanding these interactions is essential for optimizing their efficacy and developing them as multi-functional therapeutic agents.

## 2. Materials and Methods

### 2.1. Chemicals and Instruments

Chemicals (purity > 98%) used for synthesis, characterization, assessment of antimicrobial, and anti-inflammatory activities of newly synthesized pyrogallol-coumarin hybrids: 4-hydroxycoumarin, 2,3,4-trihydroxybenzoic acid, 3,4,5-trihydroxybenzoic acid, hydrazine monohydrate, phosphorus oxychloride, dimethylsulfoxide (DMSO-*d*_6_), methanol, ethanol, toluene, acetone, dimethylsulfoxide, acetic and hydrochloric acid soybean lipoxygenase, sodium linoleate, and tris buffer were purchased from Merck (KGaA, Darmstadt, Germany). The starting compound 3-acetyl-4-hydroxycoumarin and hydroxybenzohydrazides were synthesized using previously established methods [16,17].

The infrared spectra were recorded with a Perkin-Elmer FT–IR spectrophotometer (KBr technique) in the range 4000–400 cm^−1^. ^1^H and ^13^C NMR NMR spectra of the newly synthesized compounds were recorded using a Varian Gemini–200 NMR spectrophotometer in DMSO-d6 as solvent. The chemical elements hydrogen, nitrogen and carbon were quantified in the samples using ECS 8020 (NC Technologies, Bussero, Italy).

### 2.2. Chemical Studies

#### Synthesis of **PCH**s

**Scheme 1:**
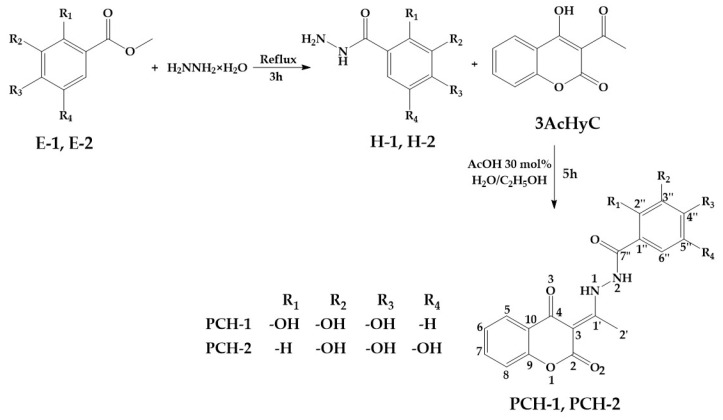
Synthesis of **PCH**s.

Compounds **PCH-1** and **PCH-2** were synthesized by reacting 3-acetyl-4-hydroxycoumarin with corresponding hydroxybenzohydrazides (**H-1** and **H-2**) [17]. The equimolar amounts of hydroxybenzohydrazide and **3AcHyC** were dissolved in a mixture of water and ethanol (1:1), and afterward, 30 mol% of acetic acid as a catalyst was added. The mixture was then agitated at reflux for a duration of 5 h at a temperature of 80 °C. The progression of the reaction was monitored using thin-layer chromatography (ethyl acetate and dichloromethane in a 2:1 ratio were employed as the eluent). After the reaction was complete, the resulting mixture was cooled, and the solid substance was gathered by passing it through a filter. Subsequently, the resulting products were purified by recrystallization using ethanol as a solvent (Scheme 1).

(*E*)-*N*′-(1-(2,4-dioxochroman-3-ylidene)ethyl)-2,3,4-trihydroxybenzohydrazide (**PCH-1**) yield: 0.382 g (77%). Calculated C_18_H_14_N_2_O_7_ (FW = 370.39)%: C, 58.38; H, 3.81; N, 7.56; O, 30.24. Found: C, 58.16; H, 3.89; N, 7.79; O, 30.16, **^1^H NMR** (DMSO–*d*_6_, 200 MHz) (δ ppm): 15.83 (s, 1H), 11.37 (s, 2H), 9.98 (s, 1H), 8.74 (s, 1H), 8.00 (dd, *J* = 7.8, 1.5 Hz, 1H), 7.73–7.61 (m, 1H), 7.40–7.21 (m, 3H), 6.46 (d, *J* = 8.8 Hz, 1H), 2.72 (s, 3H); **^13^C NMR** (DMSO–*d*_6_, 50 MHz) δ (ppm): 17.5, 95.4, 106.5, 107.7, 116.3, 119.7, 123.9, 125.7, 132.9, 134.3, 149.5, 150.9, 153.1, 161.5, 166.4, 171.0, 178,9; **IR** (KBr) υ(cm^−1^): 3469 (H–N2), 3358 (H–O4″), 3275 (H–O3″, H–O2″), 2965 (H–N1), 1668, 1613, 1602 (C=O), 1555, 1506, 1327 (C–C), 1233, 1164 (C–O).

(*E*)-*N*′-(1-(2,4-dioxochroman-3-ylidene)ethyl)-3,4,5-trihydroxybenzohydrazide (**PCH-2**) yield: 0.259 g (68%). Calculated: C_18_H_14_N_2_O_7_ (FW = 370.39)%: C, 58.38; H, 3.81; N, 7.56; O, 30.24. Found: C, 58.26; H, 3.77; N, 7.77; O, 30.56 **^1^H NMR** (DMSO–*d*_6_, 200 MHz) (δ ppm): 15.73 (s, 1H), 11.48 (s, 1H), 9.37 (s, 2H), 9.05 (s, 1H), 7.99 (dd, *J* = 7.8, 1.5 Hz, 1H), 7.80–7.55 (m, 1H), 7.32 (td, *J* = 8.3, 1.9 Hz, 2H), 6.95 (s, 2H), 2.73 (s, 3H); **^13^C NMR** (DMSO–*d*_6_, 50 MHz) δ (ppm): 17.8, 95.2, 107.5, 116.4, 119.9, 120.8, 123.9, 125.8, 134.3, 138.1, 145.8, 153.2, 161.6, 165.0, 171.4, 179.2; **IR** (KBr) υ (cm^−1^): 3500, 3470 (H–N2), 3377 (H–O4″), 3275 (H–O3″), 3197 (H–O2″), 2645 (H–N1), 1654, 1612 (C=O), 1552, 1529, 1467 (C–C), 1275 (C–O).

### 2.3. DFT Calculations

The geometries of investigated compounds were optimized using the Gaussian09 software package [26]. The resulting structures were visualized with GaussView 6.0.16 [27]. Quantum chemical calculations were performed at the B3LYP-D3BJ/6-311++G(d,p) level of theory, incorporating both polarization and diffuse functions. This theoretical approach is well-supported in the literature for accurately determining various structural parameters and was selected according to our previous research on structural parameters for similar compounds [28,29,30]. To mimic experimental conditions, the solvent effect was accounted for using the Conductor-like Polarizable Continuum Model (CPCM) with water (ε = 78.36) as solvent [31]. Obtained geometries did not possess any imaginary frequencies, indicating that they represent structures on the minima of the potential energy surface. For simulations of NMR spectral data, the *Gauge Intrinsic Atomic Orbital* GIAO method was used [32]. Chemical shifts for hydrogen and carbon atoms were obtained in regard to TMS, which was used as an internal standard in experimental work.

### 2.4. Biological Studies

#### 2.4.1. In Vitro Tests for the Assessment of Antimicrobial Activity

##### Test Microorganisms

The antimicrobial activity of **PCH-1** and **PCH-2** was tested against 13 microorganisms. The experiment included four standard strains, *Staphylococcus aureus* ATCC 6538, *Bacillus subtilis* ATCC 6633, *Proteus mirabilis* ATCC 12453, and *Pseudomonas aeruginosa* ATCC 27853; five isolates, *Staphylococcus aureus*, *Escherichia coli*, *Salmonella enterica*, *Klebsiella pneumoniae*, and *Bacillus subtilis*; and one probiotic strain *Lactobacillus plantarum* 299V. Additionally, three fungal strains were tested, including one standard strain, *Candida albicans* ATCC 10231, and two isolates, *Saccharomyces cerevisiae* and probiotic strain *Saccharomyces boulardii.*

##### Suspension Preparation

The bacterial suspensions were prepared using the direct colony method [33]. The turbidity of the initial suspension was adjusted using a densitometer (DEN-1, BioSan, Latvia). When adjusted to the turbidity of the 0.5 McFarland standard, the bacterial suspension contains approximately 10^8^ colony-forming units (CFU)/mL, and the yeast suspension contains approximately 10^6^ CFU/mL. For the experiment, the initial suspension was further diluted at a 1:100 ratio in a sterile 0.85% saline solution.

##### Microdilution Method

The antimicrobial activity was assessed by determining the minimum inhibitory concentrations (MICs) and minimum microbicidal concentrations (MMCs) using the microdilution plate method with resazurin (Alfa Aesar GmbH & Co. (KG, Karlsruhe, Germany)) [34]. For this, 96-well plates were prepared by adding 100 μL of Mueller Hinton broth (Torlak, Belgrade, Serbia) for bacterial tests and Sabouraud broth (Torlak, Belgrade, Serbia) for yeast into each well. The tested **PCH-1** and **PCH-2** were dissolved in 10% dimethyl sulfoxide (DMSO) and then diluted into sterile distilled water. A 100 μL sample from the stock solution of the tested compound was then added to the first row of the plate, followed by twofold serial dilutions using a multichannel pipette. This produced a concentration range from 2.69 to 0.0025 µmol/L. The method is described in detail in the reported paper [35].

Tetracycline (Merck KGaA, Darmstadt, Germany) and fluconazole (Pfizer, New York, NY, USA) served as positive controls, while 10% DMSO was used as a solvent control, showing no inhibitory effect on microorganism growth. Each test setup included growth control and sterility control. All tests were conducted in duplicate, yielding consistent MIC values. Minimum microbicidal concentrations (MMCs) were determined as described in detail in the reported paper [35].

#### 2.4.2. Determination of Antibiofilm Activity

##### Biofilm Formation of Tested Bacteria

The ability of *S. aureus* ATCC 6538, *P. mirabilis* ATCC 12453, and *P. aeruginosa* ATCC 27853 to form biofilms was assayed as described by O’Toole et al. (2000) [36], with some modifications. Briefly, tissue culture 96-well microtiter plates (Sarstedt, Germany) were prepared by dispensing 100 µL of nutrient broth (i.e., Mueller Hinton broth) into each well. Then, 10 µL of spore suspension (1.0 McFarland for Gram-positive bacteria and 0.5 McFarland for Gram-negative bacteria) was added to each row. To encourage biofilm formation, all plates were incubated at 37 °C for 48 h, after which the content of each well was emptied and washed twice with 200 µL of sterile 0.85% saline to remove non-adherent cells. The biofilms formed were fixed by adding 100 µL of methanol until it evaporated completely, then stained with 0.1% (*w*/*v*) crystal violet (Fluka AG, Buchs, Switzerland) and left at room temperature for 20 min. The excess stain was washed away with deionized water, followed by 100 mL of 96% ethanol. The optical densities (ODs) of the stained biofilms were measured with an ELISA plate reader (RT-2100C, Rayto, Shenzhen, China) at 630 nm. Control wells containing only broth were included to verify sterility and prevent nonspecific media binding. To account for background absorbance, OD readings from sterile medium, fixative, and dye were averaged and subtracted from all test measurements. All tests were conducted in duplicate.

##### The Inhibition of Biofilm Formation by **PCH-1** and **PCH-2**

The tissue culture 96-well microtiter plates (Sarstedt, Germany) were prepared by dispensing 100 µL of nutrient broth (i.e., Mueller Hinton broth) into each well. From the stock solutions of tested **PCH-1** and **PCH-2** (at a concentration of 5.4 µmol/L), 100 µL was added to the first row of the microtiter plate. Twofold serial dilutions were then performed, followed by the addition of 10 µL of bacterial suspension to each well. The turbidity of the bacterial suspension was 0.5 McFarland for Gram-negative bacteria and 1.0 McFarland for Gram-positive bacteria. The microtiter plates prepared in this way were incubated at 37 °C for 48 h. The remaining steps of the experiment were carried out as outlined below. The biofilm inhibitory concentration required to reduce biofilm coverage by 50% (BIC_50_) was defined as the lowest concentration of tested **PCH-1** and **PCH-2** that showed 50% inhibition of biofilm formation. The biofilm inhibitory concentration required to reduce biofilm coverage by 90% (BIC_90_) was defined as the lowest concentration of tested **PCH-1** and **PCH-2** that showed 90% inhibition of biofilm formation [37].

Only broth or broth with tested **PCH-1** and **PCH-2** served as controls to check sterility and nonspecific binding of media. To compensate for background absorbance, OD readings from sterile medium, extracts, fixative, and dye were averaged and subtracted from all test values. All tests were performed in duplicate. Tetracycline dissolved in a nutrient-liquid medium was used as the reference compound.

### 2.5. In Vitro Tests for the Assessment of Anti-Inflammatory Activity

#### LOX Inhibition Assay

Soybean lipoxygenase (Type I-B) is a very often used enzyme for the investigation of anti-inflammatory properties of compounds [38]. The LOX inhibition assay measures the rate of oxidation of linoleic acid by lipoxygenase, which results in the formation of a colorless product that can be detected spectrophotometrically. This enzyme catalyzes the conversion of linoleic acid into hydroperoxy fatty acids and results in increased absorbance at 234 nm. The tested compounds dissolved in DMSO were incubated at room temperature with sodium linoleate (3 mM in tris buffer pH = 9) and enzyme solution (5 × 10^3^ units/mL in saline). The activity for each tested compound was presented as IC_50_ value ± standard deviation (SD) of three independent measurements.

### 2.6. In Silico LOX Inhibitory Potential

#### Molecular Docking and Molecular Dynamics Simulations

To further investigate interactions between LOX and investigated coumarin derivatives, LOX-coumarin systems were subjected to molecular docking simulations. For this purpose, AutoDock4.2 software was utilized as a part of the AMDock 1.5.2 software package, and graphical user interface [39]. Charges of the metal ions inside the receptor structure were preserved, while other parameters were set to default. More data about the methodology can be found in our previous work [13,16,28]. BOVIA Discovery Studio 2022 and PyMOL implemented in AMDock software were used for visualization of the obtained results [39,40,41]. The crystal structure of LOX was obtained from the RCSB database with PDB ID: 3O8Y. Because 3O8Y does not possess an inhibitor in its structure, Zileuton, which is a well-known FDA-approved inhibitor of 5-LOX, was selected as standard in molecular docking simulations, as well as NDGA, which is known to be a good LOX inhibitor [42,43,44].

To better understand the mechanism of LOX inhibition in the specific timeframe, best binding conformations from molecular docking were subjected to molecular dynamics (MD) simulations. For this purpose, the AMBER22 software package with AmberFF was implemented [41,42,43,44,45,46]. Initial topologies, parametrization of ligand molecules, solvation of the investigated system, as well as all other inputs necessary for MD simulations, were generated through CharmmGUI webserver [47,48]. The production step was performed using the SHAKE algorithm, with Monte Carlo barostat in the NPT ensemble. Obtained results, which are collected in the topology file, were analyzed using CPPTRAJ 22 software and presented through *Root Mean Square Deviation*—RMSD, *Root Mean Square Fluctuation*—RMSF, *Radius of Gyration*—Rg, and *number of hydrogen bonds*—nHBs, describing different structural changes in the investigated systems [41,42,43,44,45,46,47,48].

## 3. Results and Discussion

### 3.1. Chemistry

The fact that gallic and pyrogallol-4-carboxylic acid are very good antioxidants and thus can play a very important role in the prevention of diseases caused by free radical damage encouraged us to carry out the synthesis of new compounds that contain a pyrogallol moiety in their structure. Compounds *(E)-N*′-(1-(2,4-dioxochroman-3-ylidene)ethyl)-2,3,4-trihydroxybenzohydrazide (**PCH-1**) and (*E*)-*N*′-(1-(2,4-dioxochroman-3-ylidene)ethyl)-3,4,5-trihydroxybenzohydrazide (**PCH-2**) were obtained in the reaction between 3-acetyl-4-hydroxycoumarin (**3AcHyC**) and trihydroxybenzohydrazide (2,3,4-trihydroxybenzohydrazide (**H-1**) and 3,4,5-trihydroxybenzohydrazide (**H-2**) using environmentally friendly solvents such as water and ethanol. After 5 h of reflux, the newly obtained products were dried and then recrystallized in pure ethanol. Newly synthesized compounds were obtained in good yields ranging from 68 to 77%. Recently, our research group proposed a mechanism for the synthesis of coumarin-hydroxybenzohydrazide [17].

### 3.2. Spectroscopic and DFT Characterization of ***PCH-1*** and ***PCH-2***

Newly synthesized **PCH**s were characterized based on NMR (^1^H and ^13^C), and IR spectroscopic methods, as well as elemental analysis. Experimental ^1^H NMR and ^13^C NMR spectra are shown in Figures S1–S4. In the ^1^H NMR spectra of **PCH-1** and **PCH-2**, singlets originating from the C2′ methyl groups at 2.72 (**PCH-1**) and 2.73 (**PCH-2**) ppm are clearly observed. These singlets are at a slightly higher chemical shift due to the proximity of numerous carbonyl and secondary amino groups, as well as sp2 hybridized carbon atoms. The aromatic protons of the coumarin and pyrogallol fragments form the second group of protons in the ^1^H NMR spectra. These protons are in the range between 6.95 and 8.07 ppm in the experimental spectra. On the other hand, the proton from the N1–H group of both compounds has the highest chemical shift (over 15.00 ppm for both compounds), which is a consequence of the stabilization and negative inductive effects of nitrogen and oxygen in the pseudo-six-membered rings. At slightly lower chemical shifts there are singlets originating from OH groups of pyrogallolic scaffold.

The carbon atom C2′ of the methyl group in both compounds has the lowest chemical shift value (17.6 ppm) (Figures S3 and S4). The aromatic carbons of the pyrogallol part give signals in the range of 106.5–149.5 ppm, while the signals of the carbon atoms of the coumarin base appear in the range of 119.7–153 ppm. Signals with the largest chemical shifts expectedly originate from sp^2^ hybridized carbon atoms in the positions C1, C4, and C7″.

Density functional theory (DFT) studies were done to corroborate the structures of the studied compounds. The selected criteria for comparing experimental with simulated spectral data are the correlation coefficient (R) and the mean absolute error (MAE) between experimental and theoretical values. The correlation coefficient indicates the strength of the linear relationship between the experimental and theoretical values, while the mean absolute error gives insight into the average magnitude of errors in predictions.

**Figure 1:**
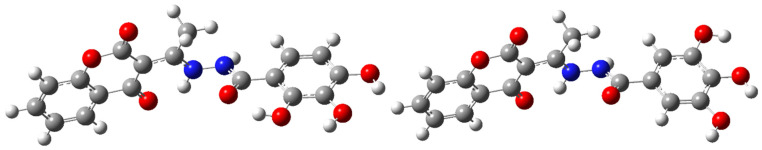
Geometries of **PCH-1** and **PCH-2** optimized at B3LYP–D3BJ/6–311++G(d,p) level of theory.

Experimental chemical shifts for newly synthesized compounds, as reported in this study, exhibit a high degree of concordance with the corresponding theoretically derived results. Namely, the correlation coefficients with the minimal average absolute error of **PCH-1** (0.997 ± 3.20) and **PCH-2** (0.992 ± 3.91) for ^13^C and **PCH-1** (0.999 ± 0.19) and **PCH-2** (0.999 ± 0.20) for ^1^H NMR (Tables S1 and S2), confirm the excellent correlation between the experimental and simulated spectral data. Considering the values of both R and MAE and because the selected DFT function provides an excellent reproduction of the experimental data for similar compounds [13,17,28], it can be confirmed that the proposed structures offer adequate representation of investigated molecules (Figure 1).

#### Analysis of Vibrational Spectra of **PCH-1** and **PCH-2**

**Figure 2:**
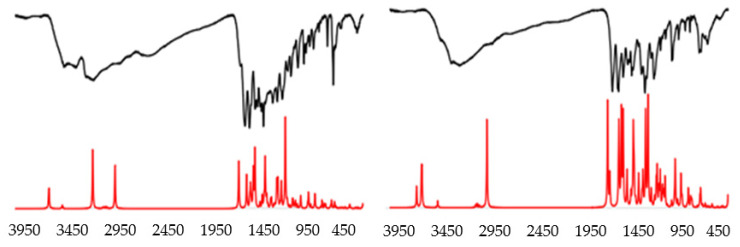
Experimental (black line) and simulated (red line, B3LYP–D3BJ/6–311++G(d,p)) vibrational spectrum for **PCH-1** (**left**) and **PCH-2** (**right**).

The vibrational spectra were recorded in the region where the wavenumber values range between 400 and 4000 cm^−1^. Theoretical spectra were simulated using the B3LYP–D3BJ functional, which in earlier cases proved to be functional and perfectly describes the vibrational IR spectra of newly synthesized benzohydrazide derivatives. Experimental vibrational spectra compared with theoretically obtained data are given in Figure 2.

In the shown IR spectra, the band positions can be divided into three regions. Bands with the highest wavenumbers are included in the first region. The N2–H stretching vibration causes very intense broadband at 3469 cm^−1^ in the experimental spectrum and around 3480 cm^−1^ in the simulated spectra. The difference in values of about 10 cm^−1^ is a consequence of the experimental conditions. Also, the hydrogen bond N2–H^……..^O7, which contributes to the stabilization of the crystal packing, is responsible for the shift of the N2–H vibration to slightly lower values of the wavenumber compared with the simulated values of the isolated molecules. O–H stretching vibrations of hydroxyl groups are very intense in the range of 3358–3275 cm^−1^. The bands originating from aromatic C–H stretching vibrations are located in a slightly lower region in the experimental spectrum at about 3000 cm^−1^. The N1–H stretching vibration has a lower wave number (2965 cm^−1^) than the N2–H stretching vibration due to stabilization by a strong intramolecular interaction in the pseudo-six-membered ring.

Next is the area of C-C, C-O stretching and C-C-H bending vibrations. The values of the wave numbers of C=O stretching vibrations decrease in the following order: C2=O (1668 cm^−1^), C7=O (1613 cm^−1^), C4=O (1602 cm^−1^). The scaled theoretical values, C2=O (1717 cm^−1^), C7″=O (1698 cm^−1^), and C4=O (1600 cm^−1^), reproduce the experimental values well. The C2=O stretching vibration has the highest wavenumber value because this group is free and undisturbed by intramolecular interactions. C7″=O vibrates at slightly lower values due to intramolecular stabilization with the N2–H group in the crystal packing. Finally, it was determined that the intensity of the C4=O vibration was reduced, and the position of the band shifted to a lower wavelength due to stabilization in the pseudo-six-membered ring. The vibrations between 1586–1333 cm^−1^ in the experimental spectrum and 1575–1359 cm^−1^ in the simulated spectrum are primarily caused by C–C–H bending vibrations. On the other hand, C–O stretching and O–C–H bending vibrations appeared at 1233 and 1164 cm^−1^ in the experimental spectra, while the corresponding signals in the simulated ones were in the range of 1234–1119 cm^−1^. The third region consists primarily of C–C–C–H, O–C–C–C, H–C–C–H, H–C–C–O, and H–C–C–H torsional modes, which located at 1043, 1036, 974, and 758 cm^−1^ in the experimental spectrum, respectively, and between 1092 and 677 cm^−1^ in the simulated spectrum.

### 3.3. Biology

#### 3.3.1. Antimicrobial Activity

The results of the in vitro antibacterial and antifungal activities of **PCH-1** and **PCH-2** determined by MICs and MMCs are shown in Table 1. A total of 13 species of microorganisms were tested, and the results were compared with the influence of tetracycline for bacteria and with fluconazole for fungi.

In this experiment, the values of the MIC and MMC ranged from 0.08 mM to 2.70 mM. The intensity of antibacterial activity depended on the type of compound and the species of bacteria. Both tested compounds (**PCH-1** and **PCH-2**) showed the best antibacterial effect on *P. mirabilis* ATCC 12453 (MIC at 0.08 mM and MMC at 0.17 mM). When comparing these two compounds, **PCH-2** showed a better antibacterial effect on *B. subtilis* ATCC 6633, *S. aureus* ATCC 25923, *S. aureus*, *L. plantarum*, *P. aeruginosa* ATCC 27853, *E. coli*, and *S. enterica* compared with other tested bacteria.

The examined fungi showed low sensitivity to **PCH-1** and **PCH-2** compared to bacterial strains (MIC and MMC in the range from 0.17 mM to 2.70 mM). The most sensitive was *C. albicans* ATCC 10231 (Table 1.)

#### 3.3.2. Antibiofilm Activity

Biofilms were quantified by measuring the absorbance of stained biofilms at 630 nm with an ELISA plate reader. The three standard bacterial species (*S. aureus* ATCC 25923, *P. mirabilis* ATCC 12453, and *P. aeruginosa* ATCC 27853) were chosen for testing because they formed the strongest biofilm among the other tested bacteria, and the tested compounds (**PCH-1** and **PCH-2**) showed a good antibacterial effect against them.

The in vitro activity of **PCH-1** and **PCH-2** on the inhibition of biofilm formation was examined. The absorbance values of controls were subtracted from the absorbance values of the tested samples (fixed and dyed) in order to compensate for background absorbance. **PCH-2** showed better activity in inhibiting biofilm formation compared with **PCH-1** for BIC_50_ values (Table 2). In fact, **PCH-1** showed no effect at the tested concentrations. Among the three tested bacteria, *P. aeruginosa* ATCC 27853 was resistant to both tested compounds (BIC_50_ and BIC_90_ at >2.70 mM). The strongest effect was noticed on the biofilm formation of *S. aureus* ATCC 25923 (BIC_50_ at 1.02 mM).

#### 3.3.3. *In Vitro* LOX Inhibition Assay

Many studies suggest that lipoxygenase-mediated products cause biological activities that lead to various diseases associated with inflammatory processes [46,47,48]. Therefore, agents that block lipoxygenase-catalyzed activity may be effective in the prevention of arthritis, allergic asthma, psoriasis, inflammatory bowel disease, cardiovascular problems, and various types of cancer [44,49,50]. Bearing in mind these facts, newly obtained **PCH**s and corresponding starting compounds (**H-1**, **H-2**, and **3AcHyC**) were subjected to investigation of their anti-LOX properties (Table 3 and Tables S3–S8, Figures S5–S10). For this purpose, the anti-LOX activities of the tested compounds at 100, 50, and 25 µM concentrations, as well as the inhibitory concentrations (IC_50_s), were determined. IC_50_ values of **PCH-1** and **PCH-2** were 38.12 μM and 34.12 μM, respectively, whereas the IC_50_ value of positive control (NDGA) was 17.27 μM. On the other hand, the inhibitory potential of precursors **H-1**, **H-2**, and **3AcHyC** was lower with IC_50_ values of 65.4, 46.0, and 57.9 µM. Also, the excellent activity of the synthesized compounds over 95% was observed at a concentration of 100 µM, while the starting compounds at that concentration exhibited activities in the range of 73.5–91.9%. The results showed that both derivatives exhibited significant inhibitory activity against the target enzyme. All this indicates a strong synergistic effect of the pyrogallol and coumarin units in the process of lipoxygenase enzyme inhibition. Due to their inhibitory effect, **PCH-1** and **PCH-2** showed potential as therapeutic agents for disorders that are characterized by excessive LOX activity, such as inflammation and cancer. In order to elucidate the mechanism of action of the tested compounds on lipoxygenase, an additional investigation using molecular docking and molecular dynamics techniques was carried out in this paper.

#### 3.3.4. *In Silico* LOX Inhibitory Potential Molecular Docking and Molecular Dynamics Simulations

**Figure 3:**
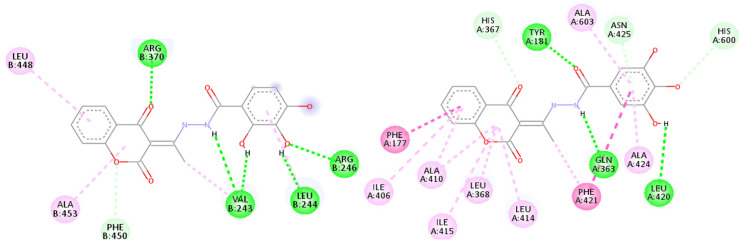
Two-dimensional representation of interactions **PCH-1** (**left**) and **PCH-2** (**right**) are forming in the active site of LOX. Conventional hydrogen bonds are presented in dark green, non-conventional hydrogen bonds are in light green, π-alkyl interactions are given in pink, while violet represents π-π T-shaped interactions.

According to the results of molecular docking simulations, the investigated pyrogallol-coumarin hybrids showed promising inhibitory potential towards LOX, correlating well with experimental data. Notably, as in the experimental work, **PCH-1**, with binding energies of −9.11 kcal/mol and an inhibitory constant of 210.05 nM, exhibited inhibitory potential similar to **PCH-2**, which has a binding energy of −8.54 kcal/mol and an inhibitory constant of 549.82 nM. The high inhibitory potential of the investigated compounds is due to numerous non-covalent interactions with amino acid residues in the protein’s active site. As shown in Figure 3, **PCH-1** forms five conventional and one unconventional hydrogen bond, while **PCH-2** forms three conventional and three unconventional hydrogen bonds, aligning with the binding energies and inhibitory constants. In comparison to Zileuton, which under the same simulation conditions shows a binding energy of −7.95 kcal/mol and an inhibitory constant of 1490 nM, and NDGA with a binding energy of −8.17 and inhibitory constant of 1030 nM, the investigated compounds show better inhibitory potential towards LOX.

To further investigate the interactions formed throughout the molecular docking simulations and explain the inhibitory potential of the investigated compounds towards LOX over a specific timeframe, MD simulations were performed. A timeframe of 50 ns was selected because the investigated systems stabilized at 10 ns.

A closer examination of Figure 3 reveals that the compounds do not adopt the same conformation inside the active sites. This is due to the additional stabilization of **PCH-1** by intramolecular hydrogen bond [17,28], preventing it from fitting inside the active site in the same way as **PCH-2**, which has one more rotatable bond (conditionally speaking). Forcing **PCH-1** into the active site using a smaller box yields the same thermodynamic binding parameters as **PCH-2**. Therefore, we chose to start from the most stable docking conformation, reflected in the parameters obtained from MD simulations.

**Figure 4:**
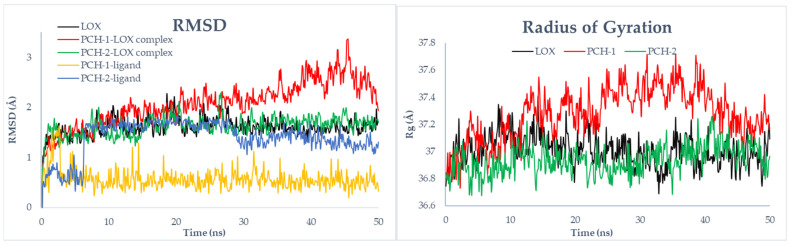
RMSD and Rg values describing the behavior of LOX-**PCH-1** and LOX-**PCH-2** systems in 50 ns in comparison to LOX.

According to the RMSD values presented in Figure 4, the binding of **PCH-2** does not significantly affect the structural characteristics of LOX. The changes induced by **PCH-1** binding are more pronounced due to the more rigid structure of the investigated compound. The rigidity of the **PCH-1** and stabilization via intramolecular hydrogen bond reflects in the RMSD values of the ligand alone. Namely, in comparison to **PCH-2**, **PCH-1** is more stable throughout the simulation period. It is obvious that conformational changes in the **PCH-1–LOX complex** system are induced by higher stabilization of **PCH-1**, while **PCH-2** is more flexible, which is obvious from the compound structure. However, it is notable that both RMSD values show low fluctuations, indicating the high stability of both investigated compounds inside the active site of the LOX. Similarly, examining Rg values (Figure 4) shows that changes in the protein’s tertiary structure are more pronounced when **PCH-1** is bound compared with **PCH-2**. However, the effects on the protein’s secondary and tertiary structure are opposite: while **PCH-1** destabilizes the protein’s structure, **PCH-2** induces additional stabilization. This difference arises because **PCH-2** forms more non-covalent interactions inside the active site, stabilizing the protein and blocking the active site, whereas **PCH-1** induces destabilization by forming strong bonds with specific amino acids, deforming the protein’s structure and rendering it inactive. Interestingly, RMSF values (Figure S11) do not indicate a change in the flexibility of amino acid residues upon binding either compound, although **PCH-1** binding slightly increases amino acid residue flexibility. The average number of hydrogen bonds also indicates that **PCH-1** (average nHB = 1.5) decreases stabilization, while **PCH-2** (average nHB = 5.4) increases stabilization of LOX (Figure S11).

It is important to note that these findings are not definitive, as there is a possibility that the investigated compounds interact with metal ions in LOX. Earlier research indicates that inhibitory activity towards LOX enzymes often involves interaction with metal ions, although this is not always the case [51,52,53]. For example, NDGA, which was used as a standard in the experimental work, exhibits its inhibitory activity towards LOX following multiple mechanisms. One way by which NDGA inhibits LOX is by interacting with metal ions in the active site, which cannot be replicated by molecular docking simulations. This is probably the main reason why the results for NDGA from the molecular docking simulations do not follow the trend of the experimentally obtained data. Similarly, baicalein exerts a comparable effect by chelating iron ions [54]. While it is understood that interactions with metal ions are not always a mechanism of LOX inhibition, such interactions could significantly influence the inhibitory potential and binding characteristics of the compounds. Therefore, further studies, including experimental validation and more detailed simulations considering metal ion interactions, are necessary to fully understand the inhibitory mechanisms of these compounds.

## 4. Conclusions

In this paper, two new pyrogallol-coumarin hybrids, **PCH-1** and **PCH-2**, were synthesized and characterized by elemental microanalysis and spectroscopic methods. Proposed structures were additionally confirmed using DFT calculations. Based on an investigation of antimicrobial activity, it can be concluded that both compounds exhibited better antibacterial than antifungal activity. The **PCH-2** showed better activity against the biofilm formation of selected bacteria compared with **PCH-1**. The anti-inflammatory activity was determined by the in vitro LOX inhibition test. The obtained experimental results show that the studied compounds have a strong inhibitory effect. In addition, by in silico investigation, it was determined that the investigated compounds form numerous hydrogen bonds with amino acid residues in the active site of the protein. These interactions contribute to the stability of the compound–protein complex and, thus, to its inhibitory effect. Because investigated compounds showed promising antimicrobial and anti-LOX potential, future research should focus on investigating mechanisms of action and assessing the compounds’ efficacy in vivo and toxicology, as well as optimizing their properties for potential clinical applications.

## Figures and Tables

**Table 1:** Antimicrobial activity of tested **PCH-1** and **PCH-2**.

Microorganisms	PCH-1		PCH-2		Tetracycline/Fluconazole	
	MIC	MMC	MIC	MMC	MIC	MMC
*B. subtilis* ATCC 6633	2.70	2.70	0.34	0.34	0.005	0.035
*B. subtilis*	1.35	1.35	1.35	1.35	0.002	0.005
*S. aureus* ATCC 25923	0.17	0.17	0.08	0.34	<0.001	0.005
*S. aureus*	2.70	2.70	1.35	1.35	<0.001	<0.001
*L. plantarum* 299V	2.70	2.70	0.67	0.67	<0.001	0.001
*K. pneumoniae*	0.17	0.34	0.17	0.34	0.009	0.07
*P. mirabilis* ATCC 12453	0.08	0.17	0.08	0.17	0.035	0.07
*P. aeruginosa* ATCC 27853	0.34	0.34	0.34	0.67	0.02	0.14
*E. coli*	0.34	0.34	0.17	0.34	0.005	0.01
*S. enterica*	0.17	0.17	0.08	0.34	0.001	0.01
*C. albicans* ATCC 10231	0.17	0.34	0.17	1.35	0.1	0.2
*S. cerevisiae*	1.35	2.70	1.35	2.70	0.025	0.025
*S.* *boulardii*	1.35	2.70	2.70	2.70	0.025	0.1

MIC—minimum inhibitory concentration; MMC—minimum microbiocidal concentration. Data are given in mM.

**Table 2:** Antibiofilm activity of tested **PCH-1** and **PCH-2**.

Tested Bacteria	PCH-1		PCH-2		Tetracycline	
	BIC_50_	BIC_90_	BIC_50_	BIC_90_	BIC_50_	BIC_90_
*S. aureus* ATCC 25923	˃2.70	˃2.70	1.02	˃2.70	0.56	>2.25
*P. mirabilis* ATCC 12453	˃2.70	˃2.70	2.15	˃2.70	1.31	>2.25
*P. aeruginosa* ATCC 27853	˃2.70	˃2.70	˃2.70	˃2.70	>2.25	>2.25

BIC_50_—biofilm inhibitory concentration required to reduce biofilm coverage by 50%. BIC_90_—biofilm inhibitory concentration required to reduce biofilm coverage by 90%. Data given as mM.

**Table 3:** In vitro investigation of anti-LOX activity for **PCHs**, their precursors, and reference compound NDGA.

Compound	*In Vitro* Lipoxygenase Inhibitory Activity			
	Anti-LOX Activity (%)			IC_50_ (µM)
	25 µM	50 µM	100 µM	
**PCH-1**	32.8 ± 2.4	65.1 ± 2.0	96.1 ± 0.7	38.1 ± 1.3
**PCH-2**	38.9 ± 3.1	71.7 ± 2.2	95.2 ± 1.0	34.1 ± 1.2
**H-1**	21.6 ± 1.4	37.8 ± 1.0	73.5 ± 0.6	65.4 ± 0.6
**H-2**	32.0 ± 0.1	56.1 ± 1.1	91.9 ± 0.2	46.0 ± 0.9
**3AcHyC**	26.4 ± 1.3	43.5 ± 3.1	81.2 ± 0.2	57.9 ± 1.6
**NDGA**	74.3 ± 0.4	/	/	17.3 ± 0.1

## Data Availability

Data are contained within the article and Supplementary Materials.

## Supplementary Materials

The following supporting information can be downloaded at: https://www.mdpi.com/article/10.3390/pharmaceutics16111472/s1, Figure S1: ^1^H NMR (200 MHz) spectrum of **PCH-1** recorded in DMSO-*d*_6_; Figure S2: ^1^H NMR (200 MHz) spectrum of **PCH-2** recorded in DMSO-*d*_6_; Figure S3: ^13^C NMR (50 MHz) spectrum of **PCH-1** recorded in DMSO-*d*_6_; Figure S4: ^13^C NMR (50 MHz) spectrum of **PCH-2** recorded in DMSO-*d*_6_; Figure S5: Inhibitory curve of **PCH-1** for in vitro LOX inhibition assay; Figure S6: Inhibitory curve of **PCH-2** for in vitro LOX inhibition assay; Figure S7: Inhibitory curve of **H-1** for in vitro LOX inhibition assay; Figure S8: Inhibitory curve of **H-2** for in vitro LOX inhibition assay; Figure S9: Inhibitory curve of **3AcHyC** for in vitro LOX inhibition assay; Figure S10: Inhibitory curve of NDGA for in vitro LOX inhibition assay; Figure S11: RMSF and nHB values describing the behavior of LOX- **PCH-1** and LOX-**PCH-2** systems in 50 ns timeframe; Table S1: Experimental and theoretical values of chemical shifts for **PCH-1** and **PCH-2** in ^1^H NMR spectra; Table S2: Experimental and theoretical values of chemical shifts for **PCH-1** and **PCH-2** in ^13^C NMR spectra; Table S3: Activity (%) and IC_50_ (µM) values of **PCH-1** for in vitro LOX inhibition assay; Table S4: Activity (%) and IC_50_ (µM) values of **PCH-2** for in vitro LOX inhibition assay; Table S5: Activity (%) and IC_50_ (µM) values of **H-1** for in vitro LOX inhibition assay; Table S6: Activity (%) and IC_50_ (µM) values **H-2** for in vitro LOX inhibition assay; Table S7: Activity (%) and IC_50_ (µM) values of **3AcHyC** for in vitro LOX inhibition assay; Table S8: Activity (%) and IC_50_ (µM) values of reference compound nordihydroguaiaretic acid (NDGA) for in vitro LOX inhibition assay.
