# Supplementary material for: Investigation of Antimicrobial and Anti-Inflammatory Efficacy of Newly Synthesized Pyrogallol-Coumarin Hybrids: In Vitro and In Silico Studies
Source: Pharmaceutics. 2024 Nov 18;16(11):1472. doi: 10.3390/pharmaceutics16111472 (PMC11597774; doi:10.3390/pharmaceutics16111472)
Supplement: Supplementary file 1 [file pharmaceutics-16-01472-s001.zip › pharmaceutics-3291651-supplementary.pdf]

Supplementary Material

Investigation of antimicrobial and anti-inflammatory efficacy of newly synthesized pyrogallol-coumarin hybrids: *in vitro* and *in silico* studies

Dušica Simijonović [1], Edina Avdović [1,*] Sandra Jovičić Milić [1], Marko Antonijević [1], Dejan Milenković [1], Katarina Marković [1], Mirjana Grujović [1], Danijela Lj. Stojković[1], Milan Dekić[2], Zoran Marković [1,2,3]

1 University of Kragujevac, Institute for Information Technologies, Department of Science, Jovana Cvijića bb, 34000 Kragujevac, Serbia; dusicachem@kg.ac.rs (D.S); sandra.jovicic@pmf.kg.ac.rs (S.J.M.); mantonijevic@uni.kg.ac.rs (M.A.); dejanm@uni.kg.ac.rs (M.M.); katarina.mladenovic@pmf.kg.ac.rs ((K.M.); mirjana.grujovic@pmf.kg.ac.rs (M.G.); danijela.stojkovic@kg.ac.rs (D.Lj.S); edina.avdovic@pmf.kg.ac.rs (E.A)

2 Department of Natural Science and Mathematics, State University of Novi Pazar, Vuka Karadžića 9 36300, Novi Pazar, Serbia; mdekic@np.ac.rs (M.D.); zmarkovic@np.ac.rs (Z.M.)

3 University of Applied Sciences Merseburg, Department of Engineering and Natural Sciences, Eberhard Leibnitz-Str. 2, 06217 Merseburg, Germany; zmarkovic@np.ac.rs (Z.M.)

* Correspondence: edina.avdovic@pmf.kg.ac.rs; Tel.: +381 34 6100195

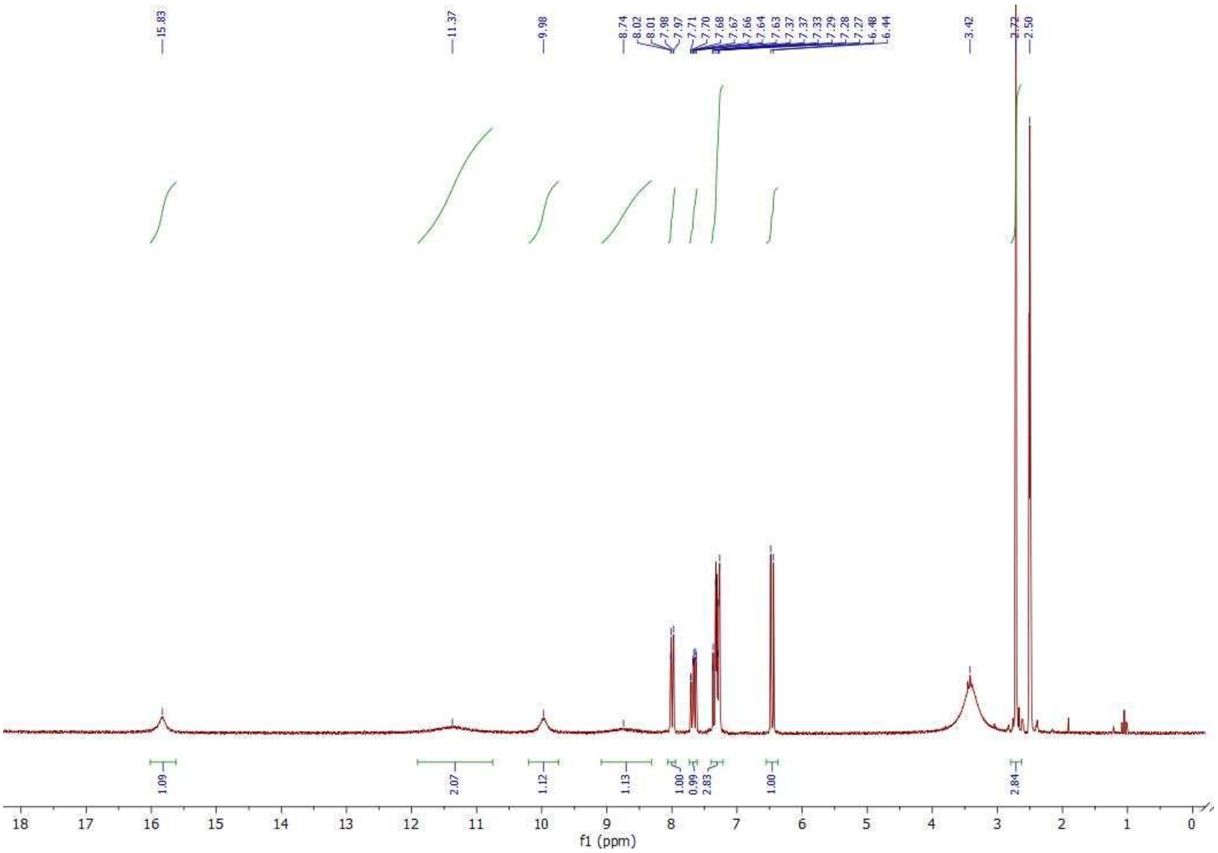

Figure S1. ^{1}H NMR (200 MHz) spectrum of **PCH-1** recorded in DMSO-d_6

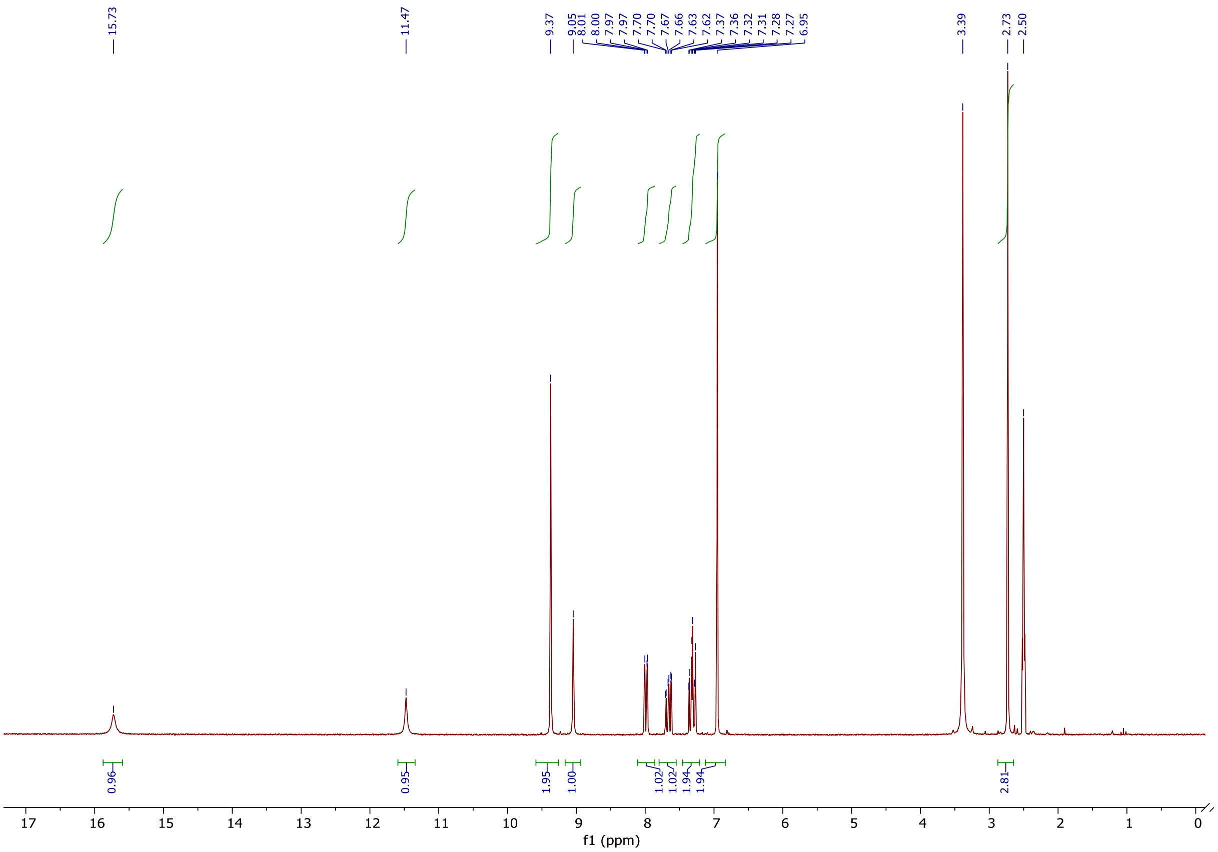

Figure S2. ^{1}H NMR (200 MHz) spectrum of **PCH-2** recorded in DMSO-d_6

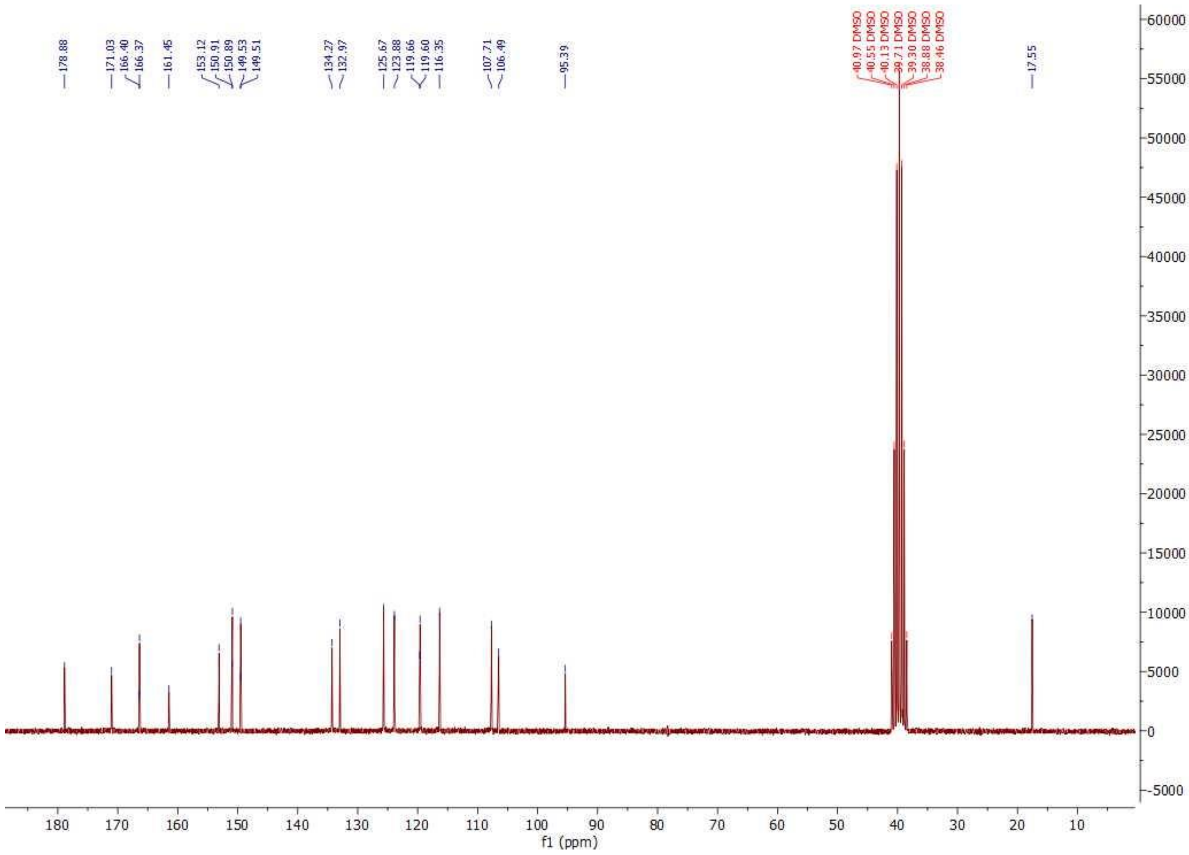

Figure S3. ^{13}C NMR (50 MHz) spectrum of **PCH-1** recorded in DMSO-d_6

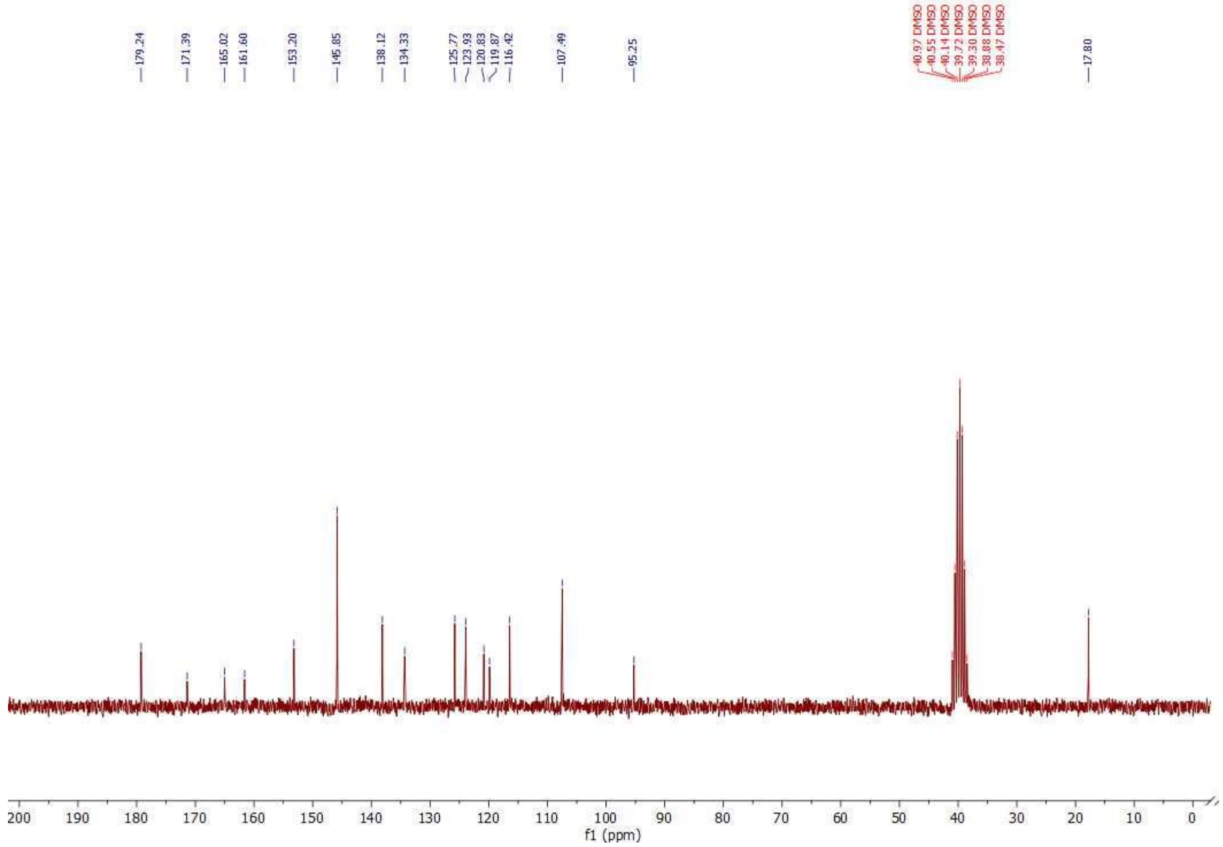

Figure S4. ^{13}C NMR (50 MHz) spectrum of **PCH-2** recorded in DMSO-d_6

Table S1. Experimental and theoretical values of chemical shifts for **PCH-1** and **PCH-2** in ^1H NMR spectra

**^1H NMR , δ (ppm)**				
Atoms	**PCH-1**		**PCH-2**	
	Experimental	Calculated	Experimental	Calculated
N1—H	15.83	15.05	15.72	15.04
N2—H	11.38	/	11.47	/
C2'—H	2.72	2.70	2.73	2.72
C5—H	8.00	8.49	7.99	8.52
C6—H	7.30	7.52	7.30	7.52
C7—H	7.67	7.96	7.66	7.92
C8—H	7.30	7.52	7.30	7.52
C2''—H/OH	11.38	/	6.95	7.19
C3''—H/OH	9.97	/	9.35	/
C4''—H/OH	8.80	/	9.46	/
C5''—H/OH	7.30	7.52	9.35	/
C6''—H	6.46	6.73	6.95	7.19
**R/MAE**	**0.999/0.19**		**0.999/0.20**	

Table S2. Experimental and theoretical values of chemical shifts for **PCH-1** and **PCH-2** in ^{13}C NMR spectra

^{13}C NMR , δ (ppm)				
Atoms	PCH-1		PCH-2	
	Experimental	Calculated	Experimental	Calculated
C2	17.6	14.1	17.6	13.9
C3	95.4	98.4	95.2	97.4
C4	171.0	182.7	171.4	182.1
C5	123.9	126.3	125.8	127.1
C6	119.7	120.0	124.0	123.2
C7	134.3	135.9	138.1	135.5
C8	116.4	115.9	119.9	116.3
C9	153.1	156.7	153.2	156.3
C10	119.6	120.0	120.8	119.7
C1'	178.9	185.1	179.4	183.4
C2'	17.6	14.1	17.8	13.9
C1"	106.5	102.1	134.4	123.0
C2"	150.9	150.6	107.5	104.7
C3"	125.7	131.6	145.8	142.0
C4"	179.5	150.4	145.8	142.0
C5"	107.7	111.8	145.8	142.0
C6"	107.7	111.8	116.4	105.1
**R/MAE**	**0.997/3.20**		**0.992/3.91**	

Table S3. Activity (%) and IC_{50} (μM) values of PCH-1 for *in vitro* LOX inhibition assay

Concentration (μM)	Activity (%)	IC_{50} (μM)
25	32.8 ± 2.4	
30	36.8 ± 2.4	
40	51.4 ± 1.8	38.1 ± 1.3
50	65.1 ± 2.0	
60	84.9 ± 0.1	

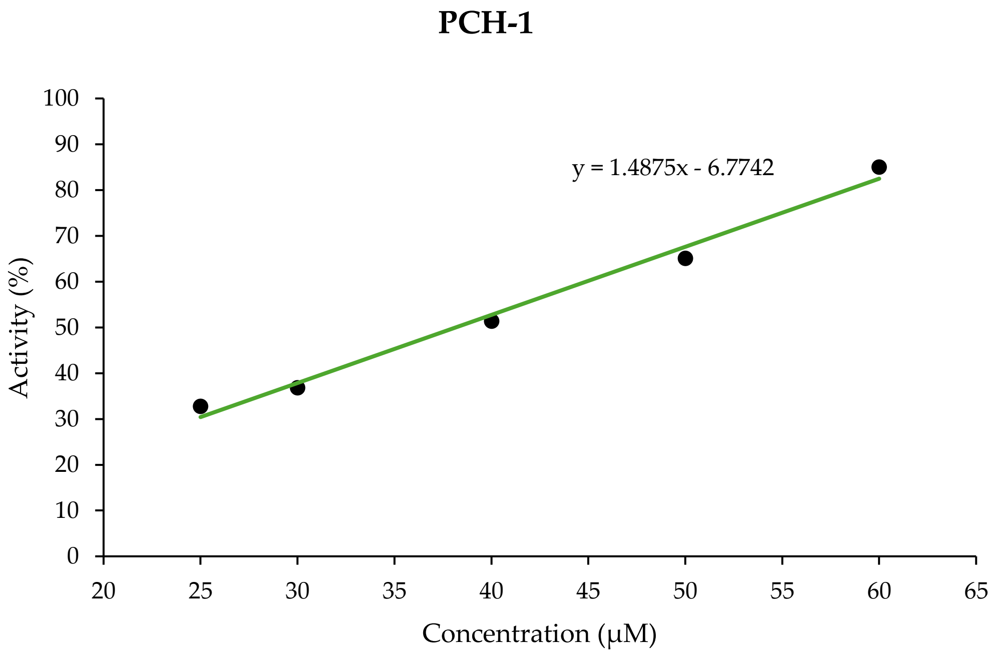

Figure S5. Inhibitory curve of PCH-1 for *in vitro* LOX inhibition assay

Table S4. Activity (%) and IC_{50} (μM) values of PCH-2 for *in vitro* LOX inhibition assay

Concentration (μM)	Activity (%)	IC_{50} (μM)
20	20.7 ± 2.7	
25	38.9 ± 3.1	
30	41.9 ± 1.4	34.1 ± 1.2
40	67.0 ± 0.9	
50	71.7 ± 2.2	

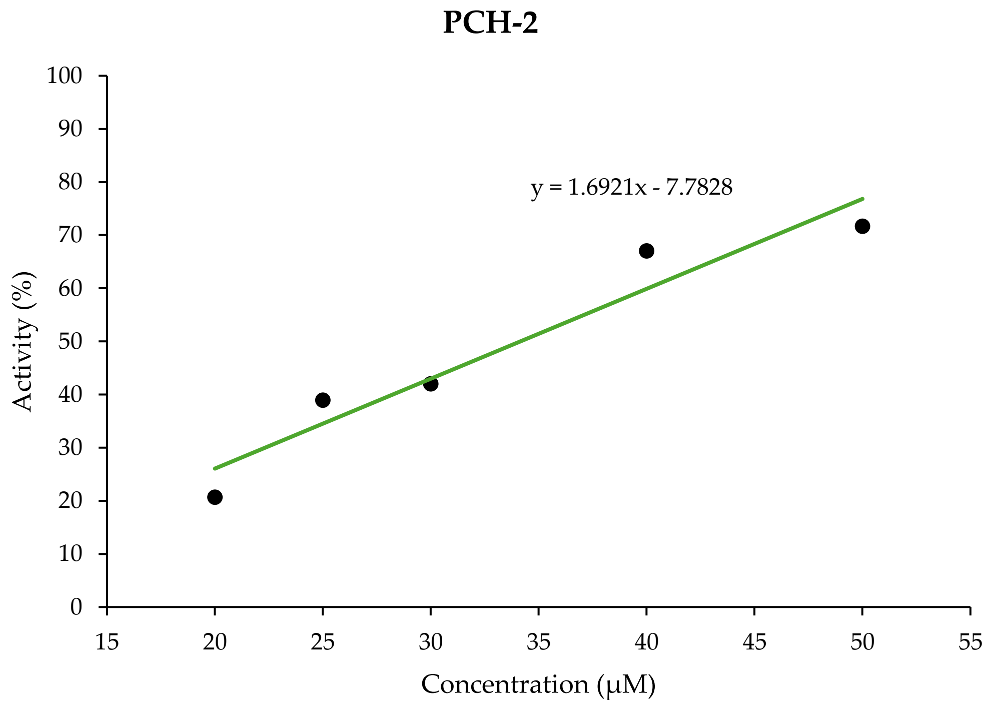

Figure S6. Inhibitory curve of PCH-2 for *in vitro* LOX inhibition assay

Table S5. Activity (%) and IC_{50} (µM) values of H-1 for *in vitro* LOX inhibition assay

Concentration (µM)	Activity (%)	IC_{50} (µM)
25	21.6 ± 1.4	
40	33.9 ± 0.8	
50	37.8 ± 1.0	65.4 ± 0.6
75	57.5 ± 1.1	
100	73.5 ± 0.6	

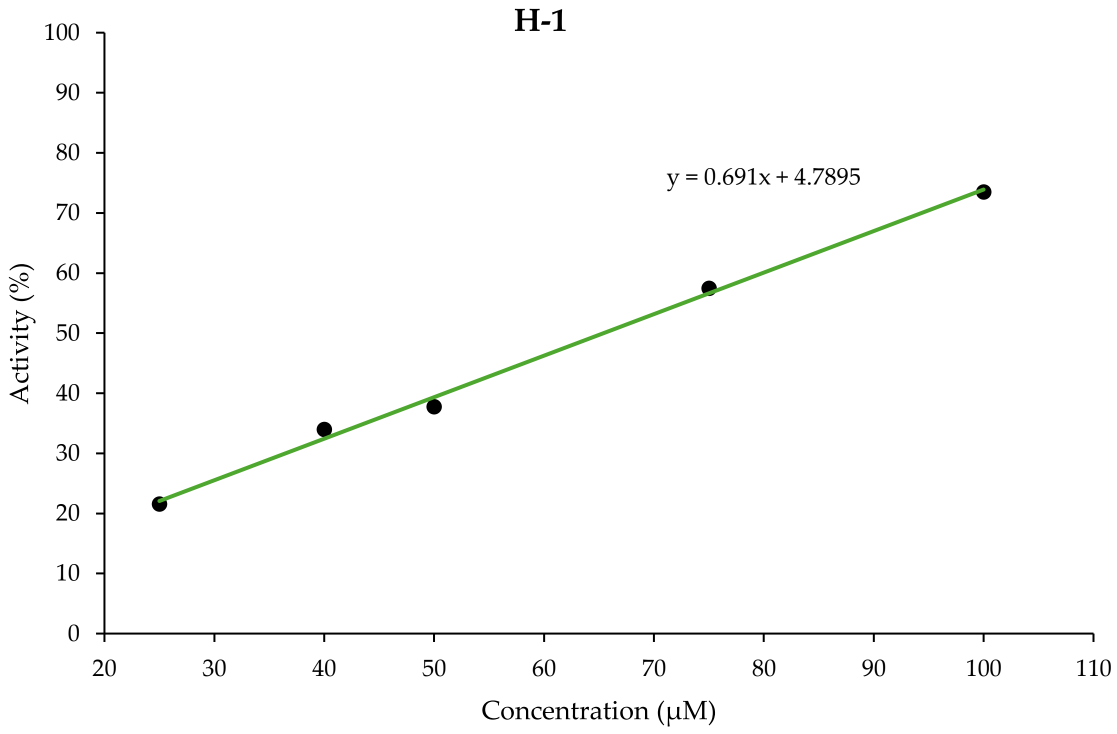

Figure S7. Inhibitory curve of H-1 for *in vitro* LOX inhibition assay

Table S6. Activity (%) and IC_{50} (μM) values H-2 for *in vitro* LOX inhibition assay

Concentration (μM)	Activity (%)	IC_{50} (μM)
25	32.0 ± 0.1	
40	44.4 ± 1.7	
50	56.0 ± 1.1	46.0 ± 0.9
75	73.4 ± 0.3	
100	91.9 ± 0.2	

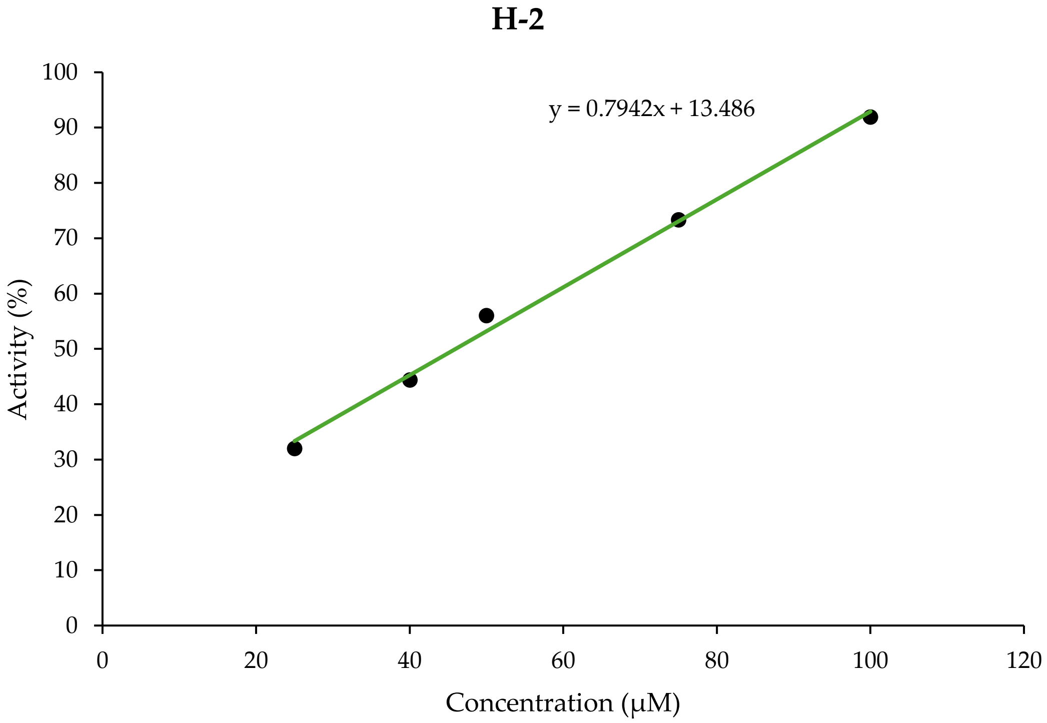

Figure S8. Inhibitory curve of H-2 for *in vitro* LOX inhibition assay

Table S7. Activity (%) and IC_{50} (μM) values of 3AcHyC for *in vitro* LOX inhibition assay

Concentration (μM)	Activity (%)	IC_{50} (μM)
25	26.4 ± 1.3	
40	35.8 ± 0.8	
50	43.5 ± 3.1	57.9 ± 1.6
75	63.4 ± 0.4	
100	81.2 ± 0.2	

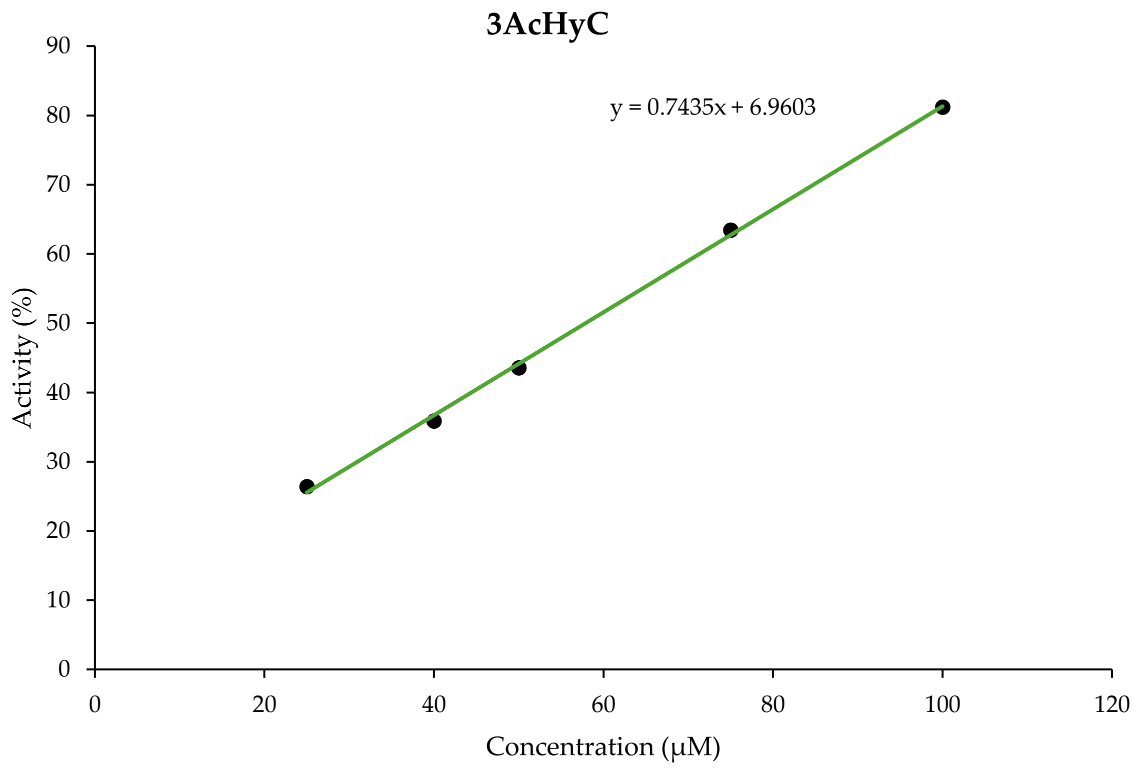

Figure S9. Inhibitory curve of 3AcHyC for *in vitro* LOX inhibition assay

Table S8. Activity (%) and IC_{50} (μM) values of reference compound nordihydroguaiaretic acid (NDGA)for *in vitro* LOX inhibition assay

Concentration (μM)	Activity (%)	IC_{50} (μM)
5	9.9 ± 0.7	
10	21.1 ± 1.6	
15	51.3 ± 1.8	17.3 ± 0.1
20	56.2 ± 0.9	
25	74.3 ± 0.4	

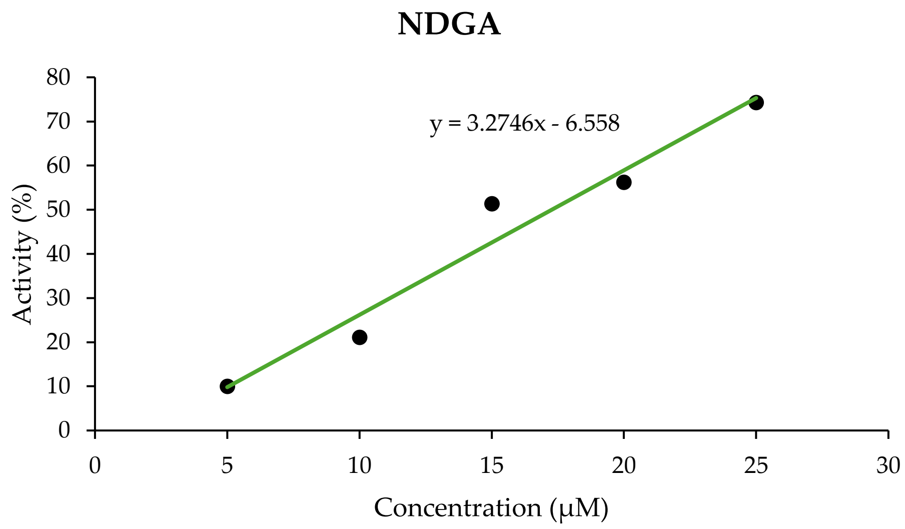

Figure S10. Inhibitory curve of NDGA for *in vitro* LOX inhibition assay

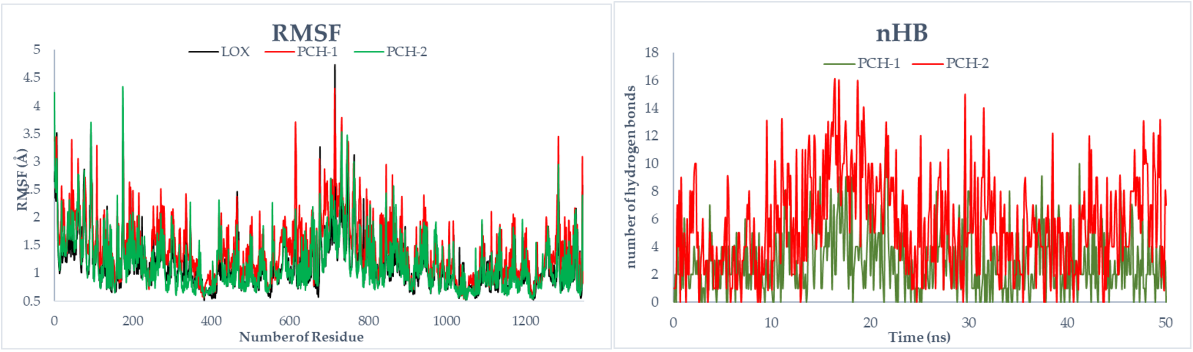

Figure S11. RMSF and nHB values describing the behaviour of LOX- **PCH-1** and LOX-**PCH-2** systems in 50 ns timeframe
